# Netrin-1 Ameliorates Postoperative Delirium-Like Behavior in Aged Mice by Suppressing Neuroinflammation and Restoring Impaired Blood-Brain Barrier Permeability

**DOI:** 10.3389/fnmol.2021.751570

**Published:** 2022-01-14

**Authors:** Ke Li, Jiayu Wang, Lei Chen, Meimei Guo, Ying Zhou, Xiaofeng Li, Mian Peng

**Affiliations:** Department of Anesthesiology, Zhongnan Hospital of Wuhan University, Wuhan, China

**Keywords:** postoperative delirium (POD), neuroinflammation, blood brain barrier, microglia cell, Netrin-1

## Abstract

Postoperative delirium (POD) is a common and serious postoperative complication in elderly patients, and its underlying mechanism is elusive and without effective therapy at present. In recent years, the neuroinflammatory hypothesis has been developed in the pathogenesis of POD, in which the damaged blood-brain barrier (BBB) plays an important role. Netrin-1 (NTN-1), an axonal guidance molecule, has been reported to have strong inflammatory regulatory and neuroprotective effects. We applied NTN-1 (45 μg/kg) to aged mice using a POD model with a simple laparotomy to assess their systemic inflammation and neuroinflammation by detecting interleukin-6 (IL-6), interleukin-10 (IL-10), and high mobility group box chromosomal protein-1 (HMGB-1) levels. We also assessed the reactive states of microglia and the permeability of the BBB by detecting cell junction proteins and the leakage of dextran. We found that a single dose of NTN-1 prophylaxis decreased the expression of IL-6 and HMGB-1 and upregulated the expression of IL-10 in the peripheral blood, hippocampus, and prefrontal cortex. Nerin-1 reduced the activation of microglial cells in the hippocampus and prefrontal cortex and improved POD-like behavior. NTN-1 also attenuated the anesthesia/surgery-induced increase in BBB permeability by upregulating the expression of tight junction-associated proteins such as ZO-1, claudin-5, and occludin. These findings confirm the anti-inflammatory and BBB protective effects of NTN-1 in an inflammatory environment *in vivo* and provide better insights into the pathophysiology and potential treatment of POD.

## Introduction

Postoperative delirium (POD) is a state of acute cerebral dysfunction characterized by fluctuating and concurrent disturbances of attention, cognition, sleep-wake rhythm, and consciousness level (Auerbach et al., 2018). It is a common complication occurring mainly within 1 week of surgery and anesthesia (Aldecoa et al., 2017). POD may lead to longer hospital stays, higher hospitalization costs, reduced life independence, increased morbidity and mortality, and long-term cognitive dysfunction, even dementia (Whitlock et al., 2011; Androsova et al., 2015). Advanced age is an independent risk factor for the incidence of POD (Marcantonio, 2011; Aldecoa et al., 2017). With the increasing aging of the global population, the number of elderly people who need surgical treatment has been growing, as has the occurrence rate of POD. Unfortunately, there are no effective treatments for this complication because of the undefined underlying pathophysiology.

In recent years, an increasing number of studies have shown that the incidence of POD is closely related to neuroinflammation (Whitlock et al., 2011; Maldonado, 2013; Vacas et al., 2013). Aseptic surgical trauma causes a homeostatic inflammatory response in the nervous system with harmful consequences when this response is dysregulated. Surgery can lead to an elevated level of pro-inflammatory cytokines in the systemic circulation, such as interleukin-6 (IL-6) and tumor necrosis factor αnfTNF-NF (Vacas et al., 2013), which are closely associated with the neuroinflammatory cascade that accompanies brain BOLD barrier failure (Rochfort and Cummins, 2015). In this case, pro-inflammatory cytokines and monocyte-derived macrophages enter, leading to the activation of glial cells, including microglia and astrocytes (Terrando et al., 2011; Hu et al., 2018). This process is mainly influenced by bone marrow-derived macrophages (BMDMs), enabling microglia/macrophages to play a two-tier role in the microenvironment of brain injury and repair (Prinz and Priller, 2014; Xiong et al., 2016). The interaction of peripheral immunity with the brain caused by systemic inflammation amplifies the inflammatory response of the central nervous system (CNS) (Matsuda et al., 2019), while the cascade of neuroinflammation leads to synaptic dysfunction and neuronal apoptosis and eventually impairs cognitive function (Girard et al., 2013; Vacas et al., 2013).

At present, possible mechanisms for the transmission of peripheral inflammatory signals to the CNS have been confirmed. First, inflammatory cytokines or macrophages derived from monocytes in peripheral blood passively diffuse into the brain through the damaged blood-brain barrier (BBB) (Terrando et al., 2011). Second, inflammatory factors enter the brain through the damaged BBB via vector-mediated active transport. Third, peripheral inflammatory signals act on the afferent branch of the vagus nerve and activate microglia in the brain to activate the inflammatory response (Cortese and Burger, 2017). In summary, changes in BBB structure and function play a very important role in this process. Yang et al. studied the effect of anesthesia and surgery on BBB permeability using the POD model previously established by our research group. The results showed that exploratory laparotomy in mice under isoflurane inhalation anesthesia caused damage to the BBB and increased permeability of the BBB to 10-kDa dextran (Yang et al., 2017), suggesting that the occurrence of POD in mice may be related to the increased permeability of the BBB due to damage. These findings suggest that POD may be associated with peripheral and neuroinflammation, BBB structural damage, and increased permeability.

Advances in the mechanisms underlying the resolution of acute inflammation have identified a new genus of pro-resolving lipid mediators called “specialized pro-resolving mediators” (SPMs) (Serhan, 2014), which can be increased *in vivo* by Netrin-1 (NTN-1) during acute self-limited inflammation (Dalli and Serhan, 2018). NTN-1 is an axonal guidance molecule involved in physiological and pathological processes such as apoptosis, inflammation, and neurogenesis in the nervous system, as well as in the lungs, heart, and kidneys. In recent years, NTN-1 has been shown to play an active regulatory role in the inflammatory process (Tang et al., 2016). It has also been demonstrated that NTN-1 can limit inflammatory response by participating in inflammatory cascades (Ly et al., 2005). In addition, NTN-1 is considered a survival factor for endothelial cells and can induce neovascularization and vascular remodeling. Overexpression of NTN-1 promotes angiogenesis and improves long-term neurological functions after ischemic stroke. Recent studies have indicated that NTN-1 retains BBB integrity in models of traumatic brain injury and experimental autoimmune encephalomyelitis (Podjaski et al., 2015; Xie et al., 2017). However, there are no reports of the role of NTN-1 in POD.

Based on these discoveries, we proposed the hypothesis that pretreatment with NTN-1 could improve the POD-like behavior of aged mice through its anti-inflammatory effect on the inflammation induced by surgical trauma. To validate this hypothesis, we assessed the effects of NTN-1 on the postoperative behavior of aged mice and inflammatory events in both the periphery nervous system and CNS. In addition, we aimed to determine whether NTN-1 prevents peripheral inflammatory factors from entering the brain by protecting the tightness of the BBB, which plays an important role in preventing peripheral inflammation from metastasizing to the CNS.

## Materials and Methods

### Animals

All procedures were approved by the Animal Ethics Committee of Zhongnan Hospital of Wuhan University, Hubei, China, and all experiments were performed in accordance with the National Institutes of Health Guidelines for the Care and Use of Laboratory Animals. Efforts were made to minimize the number of animals used. C57BL/6J female mice (18 months old, weighing 30–40 g) were purchased from Changsha Tianqin Biotechnology Co. Ltd., Changsha, China. All animals were group-housed at five per cage with free access to food and water. The temperature, humidity, and day-night cycle were maintained according to the standards established by the experimental animal laboratory at Zhongnan Hospital of Wuhan University. The mice were allowed 1 week to acclimatize to the laboratory environment before the experiment.

### Experimental Protocol

**FIGURE 1:**
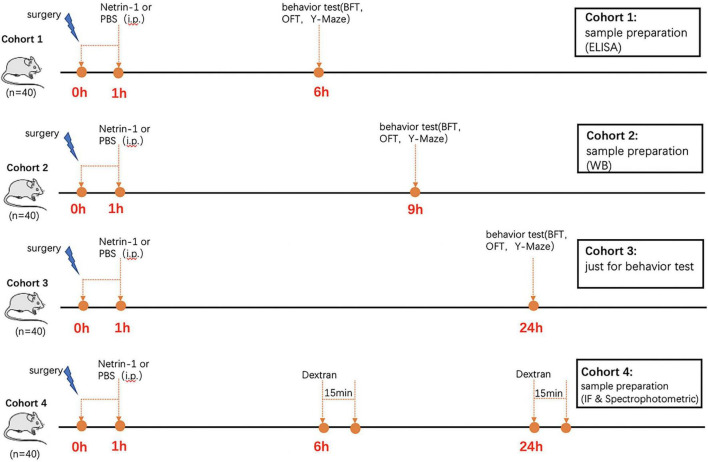
Experimental timeline. The mice were administered Netrin-1 (45 μg/kg) or vehicle control (sterile PBS) at 1 h after surgery (Day 0). The mice in Cohort 1 were used to detect behavior 6 h after surgery. Once the behavior test was completed, they were sacrificed immediately for immunofluorescence and ELISA. The mice in Cohort 2 were used to detect behavior at 9 h after surgery. Once the behavior test was completed, they were sacrificed immediately for WB. The mice in Cohort 3 were used to detect behavior 24 h after surgery. Twenty mice in Cohort 4 were injected intravenously with 100 μl 10-kDa dextran at 6 h after surgery. Fifteen minutes after injection, each mouse was prepared for immunofluorescence. Another 20 mice in Cohort 4 were injected intravenously with 100 μl 10-kDa dextran at 24 h after surgery. Fifteen minutes after injection, each mouse was prepared for spectrophotometric quantification of 10-kDa dextran.

The mice were randomly divided into four groups: control group, surgery group, surgery + NTN-1 group, and NTN-1 group. NTN-1 group (R&D Systems, 6419-N1-025) was given at 45 μg/kg in phosphate-buffered saline (PBS) and administered through the tail vein with a total volume of 200 μl at 1 h after surgery, while an equal volume of PBS was given in the control group and surgery group. The dose of NTN-1 was based on studies using a subarachnoid hemorrhage model of acute inflammation with slight modification (Xie et al., 2017). A separate cohort of mice was used for the behavior test experiments, and four cohorts are displayed on the timeline as shown in Figure 1.

### Postoperative Delirium Mouse Model

A simple laparotomy was performed under isoflurane anesthesia using the methods described in our previous studies (Lu et al., 2020). Specifically, anesthesia was induced and maintained with 1.4% isoflurane in 100% oxygen in a transparent acrylic chamber. After 15 min of induction, the mouse was removed from the chamber and placed on a heating pad to maintain a temperature between 36 and 37°C during the procedure. Isoflurane anesthesia was maintained *via* a cone device. One 16-gauge needle was inserted into the cone near the nose of the mouse to monitor the concentration of isoflurane. A longitudinal midline incision was made from the xiphoid to the 0.5-cm proximal pubic symphysis on the skin, abdominal muscles, and peritoneum. Then, the incision was sutured layer by layer with 5-0 Vicryl thread. At the end of the procedure, EMLA cream (2.5% lidocaine and 2.5% prilocaine) was applied to the incision wound and then repeated every 8 h for 1 day to treat the pain associated with the incision. The procedure lasted approximately 10 min for each mouse, and the mouse was then put back into the anesthesia chamber for up to 2 h to receive the rest of the anesthesia, consisting of 1.4% isoflurane in 100% oxygen. After recovering from the anesthesia, each mouse was returned to a home cage with available food and water. The mice in the control group and the NTN-1 group were placed in their home cages with 100% oxygen for 2 h without surgery.

### Behavioral Tests

Behavioral changes were detected using a battery of behavioral tests, including buried food tests, open-field tests, and Y-maze tests, 24 h before (baseline) surgery/anesthesia and at 6, 9, and 24 h, as described in our previous study (Peng et al., 2016). Within each group, separate cohorts were subjected to an assessment of behavior at each time point (*n* = 10 per cohort). In all tests, the apparatus was cleaned with 75% alcohol after each mouse to remove odors.

#### Buried Food Test

The buried food test was carried out as described in previous studies (Zhang et al., 2018; Lu et al., 2020) with modifications. Specifically, 2 days before the buried food test, each mouse received two pieces of sweetened cereal. On the test days, we had each mouse acclimatize for 1 h by placing the home cage with mice in the testing room. The test cage was prepared with clean padding 3 cm high in which we buried one piece of sweetened cereal below the padding. Its location was freely chosen, and it was not visible. We placed the mouse in the center of the cage and measured the latency of eating the food. The latency was defined as the time beginning when the mouse was placed in the cage and ending when the mouse uncovered the food and grasped it with its forepaws and/or teeth, which was used as a measure of organized thinking and attention. When mice found the food pellet within 5 min, they were allowed to eat the food and then were returned to their home cage. If they failed to find the pellet within 5 min, they would be retrieved and the latency was recorded as 300 s.

#### Open-Field Test

The open-field test was adapted from previous studies (Peng et al., 2016; Meng et al., 2019) with modifications. Each mouse was placed in the center of an open field chamber (40 cm × 40 cm × 40 cm) under dim light and was allowed to move freely for 5 min. The activities were automatically recorded by a video camera connected to Any-Maze animal tracking system software (Xinruan Information Technology Co. Ltd., Shanghai, China), and movement parameters were calculated by the software. The total distance moved (m), time (s) spent in the center of the open field, freezing time (s), and latency (the time in seconds for the mice to reach the location at the first attempt) to the center of the open-field were recorded and analyzed.

#### Y-Maze Test

Mice underwent testing in the Y-maze test to assess spatial learning and memory ability following surgery/anesthesia (Chen et al., 2014; Wolf et al., 2016). Specifically, the Y-maze was placed in a quiet and illuminated room and consisted of three arms (8 cm × 30 cm × 15 cm) with an angle of 120° between each arm. The three arms included the start arm, in which the mouse started to explore (always open); the novel arm, which was blocked at the first trial but opened at the second trial; and the other arm (always open). The start arm and other arm were designed randomly to avoid spatial memory error. The Y-maze test consisted of two trials separated by an intertrial interval (ITI). The first trial (training) lasted 10 min and allowed the mouse to explore the start arm and the other arm. After 2 h (for the studies 6 and 24 h after the surgery) or 4 h (for the study 9 h after surgery) of ITI, the second trial was conducted. For the second trial, the mouse was placed back in the maze in the same start arm with free access to all arms for 5 min. A video camera, which was linked to the Any-Maze animal tracking system software, was installed 60 cm above the chamber to monitor and analyze the number of entries and time spent in each arm. The time spent in and entries into the novel arms indicated the spatial recognition memory (learned behavior).

### Enzyme-Linked Immunosorbent Assay

The mouse IL-6 Enzyme-Linked Immunosorbent Assay (ELISA) kit (ELK Biotechnology, ELK1157), mouse IL-10 ELISA kit (ELK Biotechnology, ELK1143), mouse HMGB-1 ELISA kit (ELK Biotechnology, ELK1440), and mouse NTN-1 ELISA kit (CUSABIO, CSB-EL016127MO) were used to evaluate peripheral or central inflammation and the levels of NTN-1 in the brain tissue at 6 h postoperatively.

### Western Blot Analysis

The hippocampus and prefrontal cortex of the mice were harvested 9 h after surgery. Anti-ZO-1 (1:500, Abcam, ab96587), anti-occludin (1:2,000, Abcam, ab167161), and anti-claudin-5 (1:500, Biorbyt, orb214680) antibodies were used to detect the expression of tight junction (TJ)-associated proteins in the hippocampus and prefrontal cortex. Anti-β-actin (1:10,000, TDY Biotech, ab37168) was used to normalize and control for loading differences in protein levels. The bands were measured using image analysis software (AlphaEaseFC software), and changes in protein levels were presented as folds of those in the control group.

### Blood-Brain Barrier Permeability Assay

Dextran was used to measure BBB permeability as described in previous studies with modifications (Kutuzov et al., 2018; Bernstein et al., 2020). Specifically, 6 h after surgery, each mouse was injected intravenously with 100 μl 10-kDa dextran Texas Red lysine fixable (4 mg/ml, Invitrogen, D1863). Fifteen minutes after injection, each mouse was anesthetized with 1.4% isoflurane and decapitated. Brain tissue was harvested and fixed with 4% paraformaldehyde overnight at 4°C, cryopreserved in 30% sucrose, and frozen in TissueTek OCT (Sakura). Frozen sections measuring 20 μm were collected and postfixed in 4% PFA at room temperature (20–25°C) for 15 min, washed in PBS and blocked with 10% goat serum (Boster Biologic Technology, China) for 2 h, then permeabilized with 0.5% Triton X-100 and incubated with isolectin B4 (20 μg/ml, I21411, Molecular Probes, San Francisco, CA, United States) for immunostaining to visualize the blood vessels. A Zeiss LSM 510 META microscope was used to detect the fluorescence images of the injected tracer and isolectin under a 40 × objective lens. For each mouse, 20 images of 10 different slices of the hippocampus and prefrontal cortex were randomly selected, and the level of dextran found outside the vessels was analyzed using ImageJ (NIH).

Spectrophotometric quantification of 10-kDa dextran Texas Red from extracts of the hippocampus and prefrontal cortex was carried out at 24 h after surgery. Specifically, each mouse was injected intravenously with 100 μl 10-kDa dextran Texas Red lysine fixable (4 mg/ml, Invitrogen, D1863) 24 h postoperatively. Fifteen minutes after injection, each mouse was deeply anesthetized and perfused with transcranial PBS (150 ml for 5 min). Then, the mice were decapitated, and the hippocampus and prefrontal cortex were harvested. Then, we used 1% Triton X-100 in PBS to homogenize the brain tissue (100 μl/100 mg brain tissue). The tissue lysates were centrifuged at 16,000 rpm for 20 min, and the fluorescence of the supernatant was measured on a POLAR star Omega fluorometer (BMG Labtech) (ex/em 595/615 nm).

### Immunofluorescence

Twenty-four hours after surgery, each mouse was anesthetized with 1.4% isoflurane and perfused transcranially with ice-cold 0.1 M PBS followed by 4% PFA in 0.1 M PBS at pH 7.4. The brains were harvested and fixed in 4% PFA in 0.1 M PBS at 4°C, cryoprotected in 30% sucrose for 72 h, frozen in TissueTek OCT (Sakura), and cut sequentially to 20 μm. After washing in PBS and permeabilization in 0.5% Triton X-100, the sections were blocked with 10% goat serum for 2 h at room temperature to block non-specific binding and washed again in PBS. Then, the sections were incubated with rabbit anti-Iba-1 primary antibody (1:200, Abcam, ab178847) at 4°C overnight. After washing, the sections were incubated with secondary antibody (goat anti-rabbit) conjugated with Alexa Fluor dye 488 from Invitrogen (1:500) at room temperature for 2 h in the dark. Immunolabeled sections were coverslipped with 40,6-diamidino-2-phenylindole (DAPI; Invitrogen) and analyzed using a microscope (Olympus, Tokyo, Japan) equipped with an imaging system. Five high magnifications were chosen in three non-overlapping fields randomly acquired in the hippocampus and prefrontal cortex subregions using a counting frame size of 0.4 mm^2^. The images were processed, and the area of the microglia was quantified using ImageJ software (NIH). The area of the selected cells was converted into immunoreactivity, which was calculated as the percentage area density, defined as the number of pixels (positively stained area) divided by the total number of pixels (sum of positively and negatively stained areas) in the imaged field.

### Statistical Analysis

Statistical analysis was performed using SPSS version 23.0 (IBM, New York, NY, United States) or GraphPad Prism 6 (GraphPad, New York, NY, United States). The normality of the data was analyzed using the Shapiro-Wilk test, and the data were found to be normally distributed. The quantitative data are expressed as mean ± standard error of the mean (SEM), with the error bars indicating the SEM. Different groups were compared using a one-way ANOVA, followed by the Bonferroni *post hoc* test. We evaluated behavior by calculating a composite Z-score for each mouse. Specifically, the composite Z-score for the mouse was calculated as the sum of the values of 6 Z-scores (latency to eat food, time spent in the center, latency to the center, freezing time, entries to the novel arm, and duration in the novel arm) normalized with the SD for that sum in the controls. Given that the reduction (rather than increase) in time spent in the center and freezing time (open-field test) and the reduction in duration and entries to the novel arm (Y-maze test) indicate impairment of behavior, we multiplied the Z-score values representing these behaviors by one prior to calculating the composite Z-score using these values. The nature of the hypothesis testing was twofold. A value of *p* < 0.05 was considered statistically significant.

## Results

### Administration of Exogenous Netrin-1 Improves Postoperative Delirium-Like Behavior Induced by Surgery/Anesthesia in Aged Mice

**FIGURE 2:**
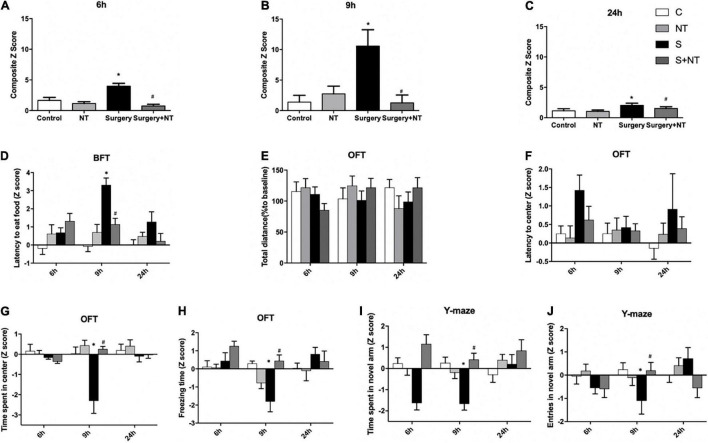
Exogenous recombinant human Netrin-1 (rh-NTN-1) improved POD-like behavior in surgical mice. **(A–C)** Changes in composite Z-scores after rh-NTN-1 treatments *via* tail vein injection at 6, 9, and 24 h postoperatively. **(E,F)** Surgery/anesthesia had no influence on latency to the center or total distance. **(D)** Surgery/anesthesia increased the latency to eat food, while exogenous rh-NTN-1 significantly decreased the latency to eat food at 9 h postoperatively. **(G–J)** Surgery/anesthesia decreased the Z-scores, while exogenous rh-NTN-1 significantly increased the Z-scores at 9 h postoperatively. The data are presented as mean ± standard error of the mean for each group (*n* = 10 per cohort). *p < 0.05 vs. the control group, *^#^p* < 0.05 vs. the surgery group.

To assess whether surgery/anesthesia affects the general and cognitive behavior of aged mice in this study, we performed a battery of behavioral tests with the food burial test, open-field test, and Y-maze test 24 h before surgery and 6, 9, and 24 h after surgery, as we previously reported (Peng et al., 2016; Lu et al., 2020). Composite Z-scores for each of the 40 mice in the four groups were calculated at 6, 9, and 24 h after surgery (*p* < 0.05, Figures 2A–C).

First, we performed a buried food test to explore whether surgery/anesthesia affected the ability of mice to associate odorants with food rewards (Yang and Crawley, 2009). The latency to eat food was markedly increased in the surgery group compared to the control group at 9 h after surgery (*p* < 0.01, Figure 2D), while administration of NTN-1 improved the impaired ability to find and eat food induced by surgery/anesthesia (*p* < 0.05, Figure 2D). No significant changes were observed between the NTN-1 group and the control group. Surgery/anesthesia-induced impairment in the ability of mice to search for and eat food suggests that surgery/anesthesia might cause the mice to develop changes in behavior (e.g., inattention, disorganized thinking, and altered level of consciousness) associated with delirium.

Then, we executed the open-field test to examine the locomotor ability and exploratory behavior of mice. There were no significant differences in the total distance traveled by mice between the four groups at the three time points after surgery, thus indicating that surgery/anesthesia did not affect the motor function of aged mice (Figure 2E). There were no significant differences in latency to the center between the four groups (Figure 2F). Surgery/anesthesia significantly decreased the time spent in the center at 9 h after surgery (*p* < 0.05, Figure 2G), and preemptive administration of NTN-1 ameliorated this phenomenon at 9 h after surgery (*p* < 0.05, Figure 2G). In addition, surgery/anesthesia significantly decreased the freezing time at 9 h after surgery (*p* < 0.05, Figure 2H), while preoperative treatment with NTN-1 increased the freezing time at 9 h after surgery (*p* < 0.05, Figure 2H). It is worth noting that NTN-1 administration did not change these parameters compared with the control condition (Figures 2G,H). These findings suggest that surgery/anesthesia altered the natural behavior of the mice, such as anxiety (time spent in the center) and natural reactions (freezing time).

Finally, we conducted a Y-maze to assess spatial memory in aged mice as previously validated (Wheelan et al., 2015). Surgery/anesthesia significantly reduced both the duration in the novel arm at 9 h after surgery (*p* < 0.05, Figure 2I) and the number of entries to the novel arm at 9 h after surgery (*p* < 0.05, Figure 2J) as compared with the control condition. Pretreatment with NTN-1 increased the number of entries to the novel arm and duration in the novel arm at 9 h after surgery (*p* < 0.05, Figures 2I,J). However, NTN-1 administration alone did not affect the performance of aged mice in the Y-maze test at 9 h after surgery (Figures 2I,J).

Taken together, no significant changes were observed between the NTN-1 group and the control group, but prophylaxis with NTN-1 attenuated the impairment of POD behavior of aged mice caused by surgery/anesthesia in a fluctuating way.

### Netrin-1 Regulates the Expression of Inflammatory Cytokines After Surgery

**FIGURE 3:**
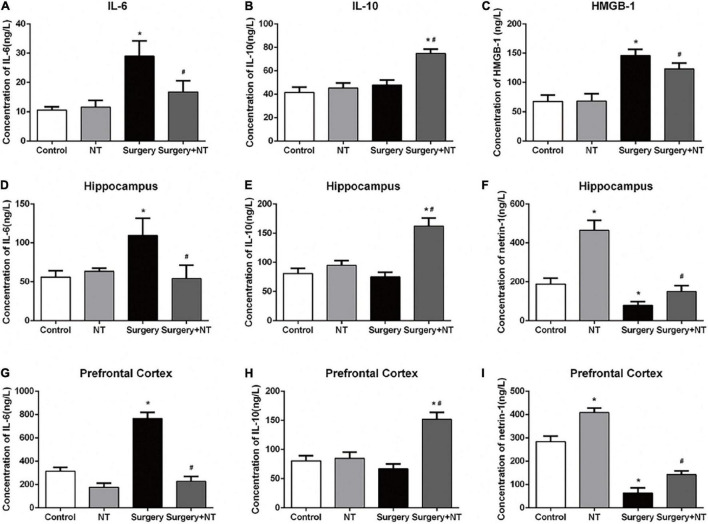
Exogenous recombinant human Netrin-1 decreased the levels of pro-inflammatory cytokines and increased the levels of anti-inflammatory cytokines in the sera, prefrontal cortex, and hippocampus of surgical mice. **(A–C)** Expression levels of IL-6, IL-10, and HMGB-1 in serum at 6 h after surgery/anesthesia. **(D–F)** ELISA was used to detect the expression of IL-6, IL-10, and Netrin-1 in the hippocampus at 6 h postoperatively. **(G–I)** Altered expression of IL-6, IL-10, and Netrin-1 in the prefrontal cortex at 6 h postoperatively. The data are presented as mean ± standard error of the mean for each group (*n* = 5 per cohort). *p < 0.05 vs. the control group, #*p* < 0.05 vs. the surgery group.

To evaluate the effects of NTN-1 on systemic inflammation, we first measured the changes in IL-6, IL-10, and HMGB-1 in blood plasma at 6 h after surgery (Yang et al., 2017). Surgery/anesthesia significantly increased the levels of IL-6 and HMGB-1 (*p* < 0.05, Figures 3A,C) but did not change the expression of IL-10 after surgery (*p* > 0.05, Figure 3B). Although a single dose of NTN-1 did not completely reverse the increase in pro-inflammatory cytokines to the control condition, it markedly reduced the levels of IL-6 and HMGB-1 after surgery (*p* < 0.05, Figures 3A,C). In addition, pretreatment with NTN-1 increased the expression of IL-10, a crucial cytokine during the resolution phase of inflammation after surgery (*p* < 0.05, Figure 3B). Second, we measured these cytokines in the hippocampus and prefrontal cortex, which are two key brain regions related to the memory network (Preston and Eichenbaum, 2013; Place et al., 2016), to evaluate the effects of NTN-1 on neuroinflammation at 6 h after surgery. Surgery/anesthesia induced a marked increase in the expression of IL-6 after surgery in both the hippocampus and the prefrontal cortex compared with the control condition (*p* < 0.05, Figures 3D,G). Pretreatment with NTN-1 significantly decreased the expression of IL-6 in these brain regions compared with that in the surgery group (*p* < 0.05, Figures 3D,G). In addition, pretreatment with NTN-1 increased the expression of IL-10 not only in the hippocampus after surgery (*p* < 0.05, Figure 3E) but also in the prefrontal cortex after surgery (*p* < 0.05, Figure 3H).

### Surgery/Anesthesia Decreases Endogenous Netrin-1 in the Hippocampus and Prefrontal Cortex in Aged Mice

To investigate whether endogenous NTN-1 was involved in the anti-inflammatory and neuroprotective effects, we measured the changes in endogenous NTN-1 in the hippocampus and prefrontal cortex at 6 h after surgery. Our results suggested that surgery/anesthesia significantly decreased the level of NTN-1 in the hippocampus and prefrontal cortex after surgery (*p* < 0.05, Figures 3F,I).

### Netrin-1 Prevents Neuroinflammation in the Hippocampus and Prefrontal Cortex

**FIGURE 4:**
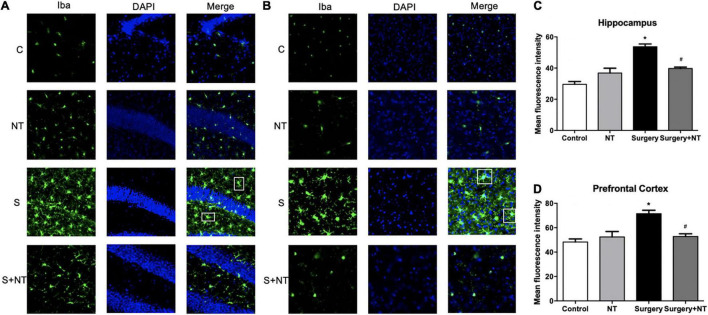
Exogenous recombinant human Netrin-1 decreased the activation of microglia in surgical mice. Activation of microglia in the hippocampus **(A)** and prefrontal cortex **(B)** 24 h postoperatively. **(C,D)** Mean fluorescence intensity in the hippocampus and prefrontal cortex 24 h postoperatively. The data are presented as mean ± standard error of the mean for each group (*n* = 3 per cohort). *p < 0.05 vs. the control group, *^#^p* < 0.05 vs. the surgery group.

We measured the changes in immunoreactivity of Iba-1 in the hippocampus and prefrontal cortex to assess the reactive states of the microglia, which represent the major pathological manifestation of neuroinflammation (Norden et al., 2016; Joshi et al., 2019). NTN-1 attenuated microglial activation by changing the expression of Iba-1. Surgery induced the amoeba-like morphology of microglia and increased the Iba-1 immunoreactivity area in the hippocampus and prefrontal cortex compared with the control condition (*p* < 0.05, Figures 4A–D), while preemptive administration of NTN-1 significantly restored the ramified shape of the microglia and reduced the cellular area (*p* < 0.05, Figures 4A–D). No significant changes in Iba-1 were observed in the NTN-1 group.

### Netrin-1 Prophylaxis Alleviates the Leakage of the Blood-Brain Barrier Induced by Surgery/Anesthesia

It has been reported that destruction of the BBB is linked to delirium and perioperative neurocognitive disorders (Maldonado, 2008; Subramaniyan and Terrando, 2019). Therefore, we used a well-established dye injection trial to investigate the integrity of the BBB (Ben-Zvi et al., 2014; Yang et al., 2017) under the treatment of surgery/anesthesia with or without the administration of NTN-1.

**FIGURE 5:**
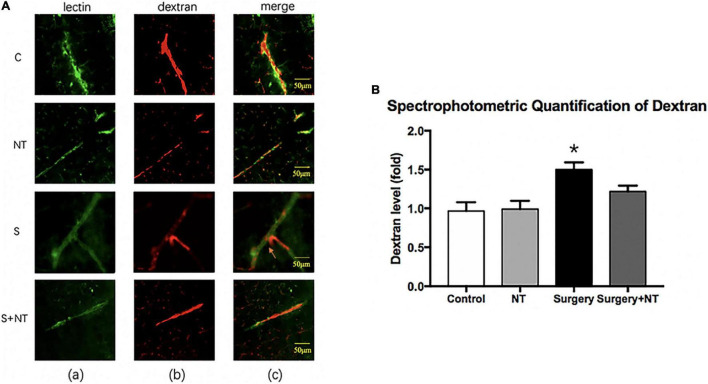
Exogenous recombinant human Netrin-1 attenuates the anesthesia/surgery-induced increase in blood-brain barrier permeability of dextran in the hippocampus of mice. **(A)** Immunostaining of blood vessels (lectin, green, Column a) and dextran (10-kDa dextran, red, Column b) in brain sections from the four groups. The red spots (non-overlapping area) in the column indicate dextran that is not inside the blood vessel (extravascular dextran). *N* = the total of 150 slides from 5 mice in each group. **(B)** Spectrophotometric quantification of brain dextran (10 kDa) levels. Anesthesia/surgery increases the brain dextran levels of mice compared with those of mice under control conditions. Treatment with rh-NTN-1 attenuates the anesthesia/surgery-induced increase in the extravascular dextran level in mice. The data are presented as mean ± standard error of the mean for each group (*n* = 5 per cohort). *p < 0.05 vs. the control group.

Immunofluorescence images showed that 10-kDa dextran was mainly confined to the vessels in the four groups. The dextran signal was detected in the brain parenchyma around the vessels of mice in the surgery group (Figure 5A). To quantitate the extravascular dextran, spectrophotometric quantification of 10-kDa dextran Texas Red from brain tissue extracts was performed. In the hippocampus, we found that surgery/anesthesia increased the level of extravascular 10-kDa dextran compared with the control condition, while NTN-1 prophylaxis decreased the leakage of dextran induced by surgery/anesthesia (*p* < 0.05, Figure 5B).

**FIGURE 6:**
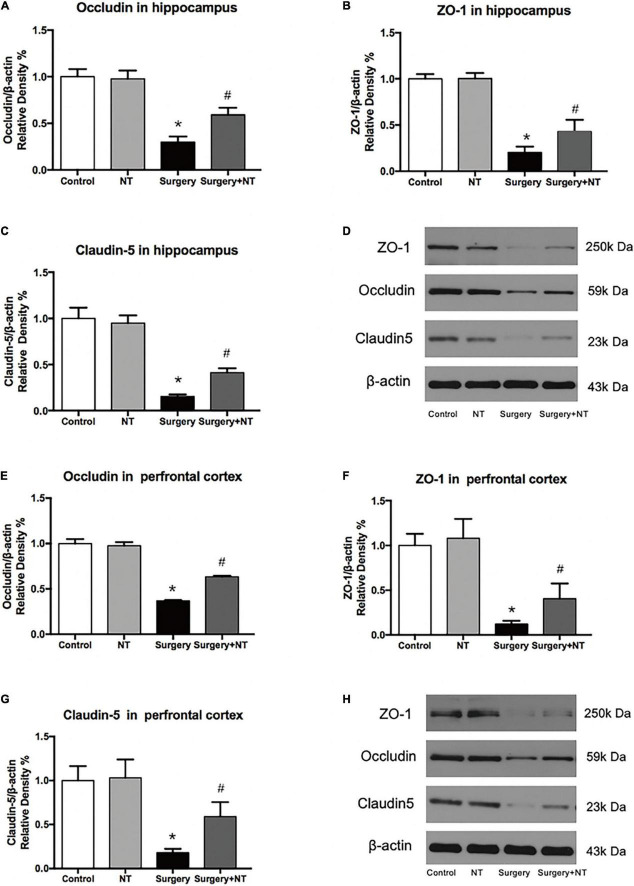
Exogenous recombinant human Netrin-1 attenuates the anesthesia/surgery-induced reduction in cell junction proteins in the hippocampus and prefrontal cortex of mice. Anesthesia/surgery reduced the protein levels of occludin **(A,D,E,H)**, ZO-1 **(B,D,F,H)**, and claudin-5 **(C,D,G,H)** in the hippocampus and prefrontal cortex of mice compared with those of mice under control conditions at 9 h after anesthesia/surgery. Treatment with rh-NTN-1 attenuated these reductions in the levels of occludin **(A,D,E,H)**, ZO-1 **(B,D,F,H)**, and claudin-5 **(C,D,G,H)**. The data are presented as mean ± standard error of the mean for each group (*n* = 5 per cohort). *p < 0.05 vs. the control group, #*p* < 0.05 vs. the surgery group.

We next examined the effects of NTN-1 on the expression of occludin, ZO-1, and claudin-5 after surgery (Figures 6D,H), which are TJ-associated proteins that maintain the integrity of the BBB (Jiao et al., 2011; Luissint et al., 2012). Using quantitative Western blot, we found that there was a marked decrease in the expression of occludin, ZO-1, and claudin-5 in both the hippocampus and the prefrontal cortex at 9 h after surgery, while pretreatment with NTN-1 significantly attenuated the reduction in these proteins (*p* < 0.05, Figures 6A–C,E–G). Preemptive administration of NTN-1 alone did not have any effects on the BBB.

## Discussion

In this study, we demonstrate that exogenous NTN-1, an axonal guidance molecule, improves postoperative POD-like behavior in aged mice through its anti-inflammatory and BBB-protecting effects. Our results show that pretreatment with NTN-1 given through the caudal vein remits the systemic inflammatory response and protects the BBB integrity after surgery/anesthesia. In addition, exogenous NTN-1 inhibited neuroinflammation in the hippocampus and prefrontal cortex according to the expression of inflammatory cytokines and reactive states of the microglia in these brain regions. To the best of our knowledge, this is the first study to report the neuroprotective effect of NTN-1 in a mouse model of POD.

Much evidence indicates that neuroinflammation plays an important role in POD. Peripheral aseptic inflammation activates the innate immune system, initiating the inflammatory process that finally leads to POD (Girard et al., 2013; Maldonado, 2013; Saxena and Maze, 2018). In an aseptic surgery environment, cellular trauma releases damage-associated molecular patterns (DAMPs), activates BMDMs by binding to Toll-like receptors (TLRs) *via* high mobility group box-1 (HMGB1), and upregulates the expression of pro-inflammatory cytokines such as IL-1, TNF-α, and IL-6 (Levy et al., 2007; Li R. L. et al., 2013). These cytokines can further activate DAMPs in positive feedback (Huang et al., 2018; Nishigaki et al., 2019) and be released into the circulation to destroy the integrity of the BBB (Li G. et al., 2013; Saxena and Maze, 2018). Our results show that NTN-1 can reduce the release of systemic pro-inflammatory factor IL-6 and increase anti-inflammatory cytokine IL-10 after surgery, which is a vital cytokine after trauma. At the same time, NTN-1 reduced the release of HMGB-1, which is passively released from cells damaged by aseptic trauma and targeted to circulating BMDMs. These findings are consistent with the potent anti-inflammatory activity of NTN-1 in many other disease models associated with inflammation, such as renal ischemia-reperfusion injury (Wang et al., 2008), acute peritonitis (Ariel et al., 2006), and acute pancreatitis (Chen et al., 2012). The migration and aggregation of the white blood cells to the inflammatory site is the central link of the whole inflammatory response. Early studies found that NTN-1 interacts with the UNC-5B receptor expressed on the surface of the white blood cells and inhibits the migration of the white blood cells (Ly et al., 2005). In Alzheimer’s disease (AD) rats (Sun et al., 2019), it has been demonstrated that NTN-1 concentrations in serum were positively correlated with the systemic expression of IL-10 (Mosser and Zhang, 2008). Moreover, in acute peritonitis and acute colitis models, NTN-1 inhibits the migration of inflammatory cells and induces the M2 polarization phenotype of macrophages (Mirakaj et al., 2011; Aherne et al., 2012). This further indicates that the changes in peripheral inflammatory factors may be related to the powerful anti-inflammatory effect of NTN-1.

A complete functional BBB is essential for proper homeostatic maintenance and perfusion of the CNS. The unique microvascular endothelial cell monolayer with inflammatory damage that forms the surface of the luminal BBB and leads to increased capillary permeability has been related to various neurological disorders ranging from ischemic stroke and traumatic brain injury to neurodegenerative disease and CNS infections (Hawkins and Davis, 2005). In addition, the neuroinflammatory cascade that typically accompanies BBB failure under these conditions has been strongly associated with elevated levels of pro-inflammatory cytokines such as TNF-α and IL-6 (Rochfort and Cummins, 2015). In models of subarachnoid hemorrhage (Xie et al., 2017), multiple sclerosis (Voortman et al., 2017), and stroke (Yu et al., 2017), NTN-1 has been shown to have a protective effect on the BBB and to improve neurocognitive function, which was also noted in our model. There is compelling evidence that exogenous NTN-1 significantly diminishes the diffusion of dextran in mouse brain-derived endothelial cells *in vitro*. NTN-1-induced barrier tightening is at least partly the consequence of NTN-induced upregulation of TJ molecules. It has been reported that the levels of both the intracellular components of the junctional complex and the transmembrane increased with the response to NTN-1. In addition, junctional proteins were enriched in lipid raft membrane microdomains after NTN-1 treatment of human brain-derived endothelial cells, and these proteins effectively interacted to form functional clusters supporting barrier integrity (Podjaski et al., 2015). Thus, NTN-1 reduces the incidence of POD by reducing the entry of peripheral inflammatory cytokines through impaired flow barriers.

In addition to alleviating the peripheral inflammatory response, NTN-1 also reduces the activation of glial cells and the expression of inflammatory cytokines in the hippocampus and prefrontal cortex. Under non-injurious conditions, microglia promote the essential functions involved in surveillance of the brain parenchyma to maintain homeostasis (Biber et al., 2007). The microglia are activated by one or more pathways following the release of pro-inflammatory cytokines by the innate immune response. The activated microglia quickly transform into a pro-inflammatory phenotype with stout morphology and enhance the production of pro-inflammatory molecules (Saxena and Maze, 2018). These pro-inflammatory cytokines and the debris released by the activated microglia can convert the astrocytes into a neurotoxic A1 reactive subtype (Hinkle et al., 2019; Li et al., 2019), which causes the astrocytes to lose their normal synaptic maintenance and phagocytosis and induces the rapid death of neurons and oligodendrocytes (Hinkle et al., 2019; Li et al., 2019). In our POD model, NTN-1 restored the morphological changes of microglia in both the hippocampus and the prefrontal cortex to their original morphology, representing the transformation of the inflammatory phenotype to the resting state, thereby changing the pro-inflammatory environment by regulating the secretion of inflammatory cytokines. Herein, it makes sense that pretreatment with NTN-1 facilitates the improvement of POD-like behavior in aged mice since the hippocampus and prefrontal cortex are responsible for shaping emotion, learning, and organizing memory (Dolleman-Van Der Weel et al., 2019; Yavas et al., 2019).

In addition, the regulation of lipid mediators by neuronal circuits might play an important role in the control of inflammation to sterile injury. The vagus nerve regulates the expression of the axonal guidance molecule NTN-1, which can increase SPM production *in vivo* if it upregulates the concentrations of RvD5 and PD1 in the exudate during acute self-limiting inflammation (Dalli and Serhan, 2018; Serhan et al., 2019). Our previous research demonstrated the anti-inflammatory and pro-resolving activities of PD1 in an inflammatory environment and identified the role of PD1 in regulating postoperative inflammation and the consequential POD-like behavior of mice (Zhou et al., 2020). In our study, compared with the control group, the concentration of endogenous NTN-1 in the hippocampus and prefrontal cortex at 6 h postoperatively was significantly reduced. This is most likely the result of endogenous NTN-1 being consumed after participating in the pro-resolution of inflammation by regulating SPM. Therefore, the neuroprotective effect of NTN-1 may be related to this mechanism. Does NTN-1 also inhibit the production of pro-inflammatory mediators by other means? It is essential to explore the underlying mechanism of NTN-1 in inflammation in further investigations.

There are several limitations to our research. First, there are several signaling pathways that have been shown to be involved in anti-inflammatory and vascular endothelial cell protection. An in-depth study of the mechanism of NTN-1 that we need to uncover will open up a novel way to prevent and treat inflammation-related lesions. Second, we have only demonstrated that exogenous prophylactic NTN-1 can improve POD by providing positive anti-inflammatory responses and protective BBB functions after surgery in elderly mice. However, how endogenous NTN-1 changes during this process has not been studied, and NTN-1 small interfering RNA (siRNA) can be used in future studies. Third, because of the frequency of fights between male mice, the occurrence of injuries, and/or the need to live alone, all these have a greater influence on behavior, and so only female mice were used in our experiments. The difference in the prevalence of POD observed between men and women is controversial in the clinic (Edlund et al., 2001; Prendergast et al., 2014; Oh et al., 2016). Perhaps gender differences affect POD development. It is worth including male mice in future studies. Fourth, NTN-1 induces neovascularization and vascular remodeling. Therefore, there may be a risk of developing cancer in older people. NTN-1 expression was elevated in melanoma compared with benign melanocytic lesions. Interference with NTN-1 expression can reduce cancer cell death and promote melanoma progression (Boussouar et al., 2020). The safety and effectiveness of NTN-1 in the clinical treatment of POD deserve further study. Finally, we only assessed the role of microglia in neuroinflammation and ignored astrocytes as important resident immune cells in the CNS, which also play an important role in neuroinflammation. In fact, there are also interactions between microglia and astrocytes in some neurodegenerative diseases (Kwon and Koh, 2020). The effect on astrocytes in POD deserves further investigation in future studies.

## Conclusion

This study determined that the administration of exogenous NTN-1 could regulate postoperative inflammation and protect the integrity of the BBB to improve POD in aged mice. These findings indicate the potential of NTN-1 as a novel therapy for POD.

## Data Availability Statement

The raw data supporting the conclusions of this article will be made available by the authors, without undue reservation.

## Ethics Statement

The animal study was reviewed and approved by Animal Ethics Committee of Zhongnan Hospital of Wuhan University.

## Author Contributions

KL and JW designed and performed the experiment, collected and analyzed the data, prepared the manuscript, and participated in the statistical analysis. MG and XL were involved in preparing the animal models and participated in interpreting the results. LC contributed to the behavioral testing. YZ was involved in the biochemical analysis. MP contributed to the study concept and design, secured funding for the project, and prepared and critically revised the manuscript. All authors reviewed the manuscript and approved the submitted version.

## Conflict of Interest

The authors declare that the research was conducted in the absence of any commercial or financial relationships that could be construed as a potential conflict of interest.

## Publisher’s Note

All claims expressed in this article are solely those of the authors and do not necessarily represent those of their affiliated organizations, or those of the publisher, the editors and the reviewers. Any product that may be evaluated in this article, or claim that may be made by its manufacturer, is not guaranteed or endorsed by the publisher.
