# Supplementary figures and images for: Netrin-1 Ameliorates Postoperative Delirium-Like Behavior in Aged Mice by Suppressing Neuroinflammation and Restoring Impaired Blood-Brain Barrier Permeability
Source: Front Mol Neurosci. 2022 Jan 14;14:751570. doi: 10.3389/fnmol.2021.751570 (PMC8797926; doi:10.3389/fnmol.2021.751570)

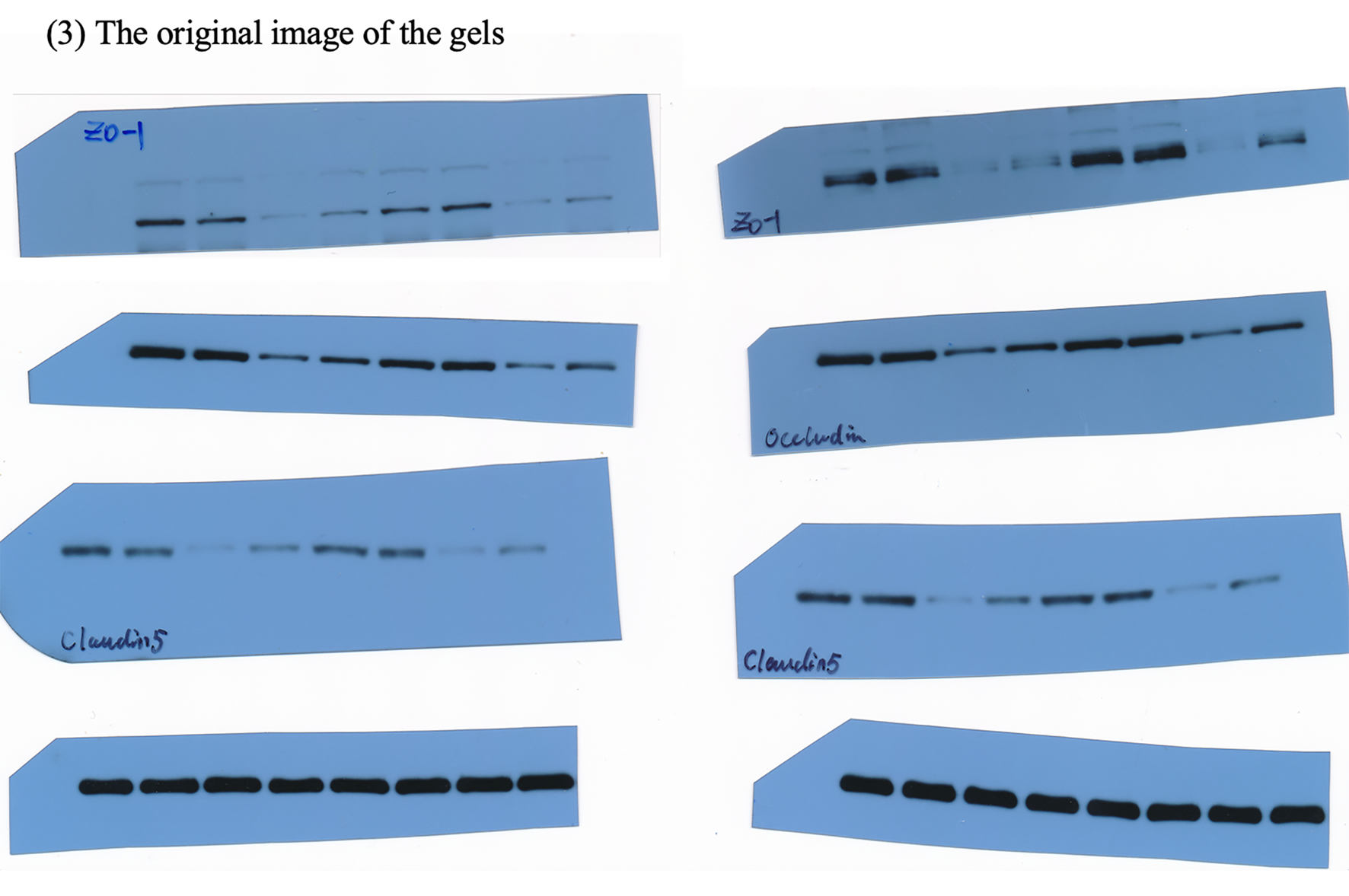

Supplement: Supplementary file 1 [file Image_1.TIF]

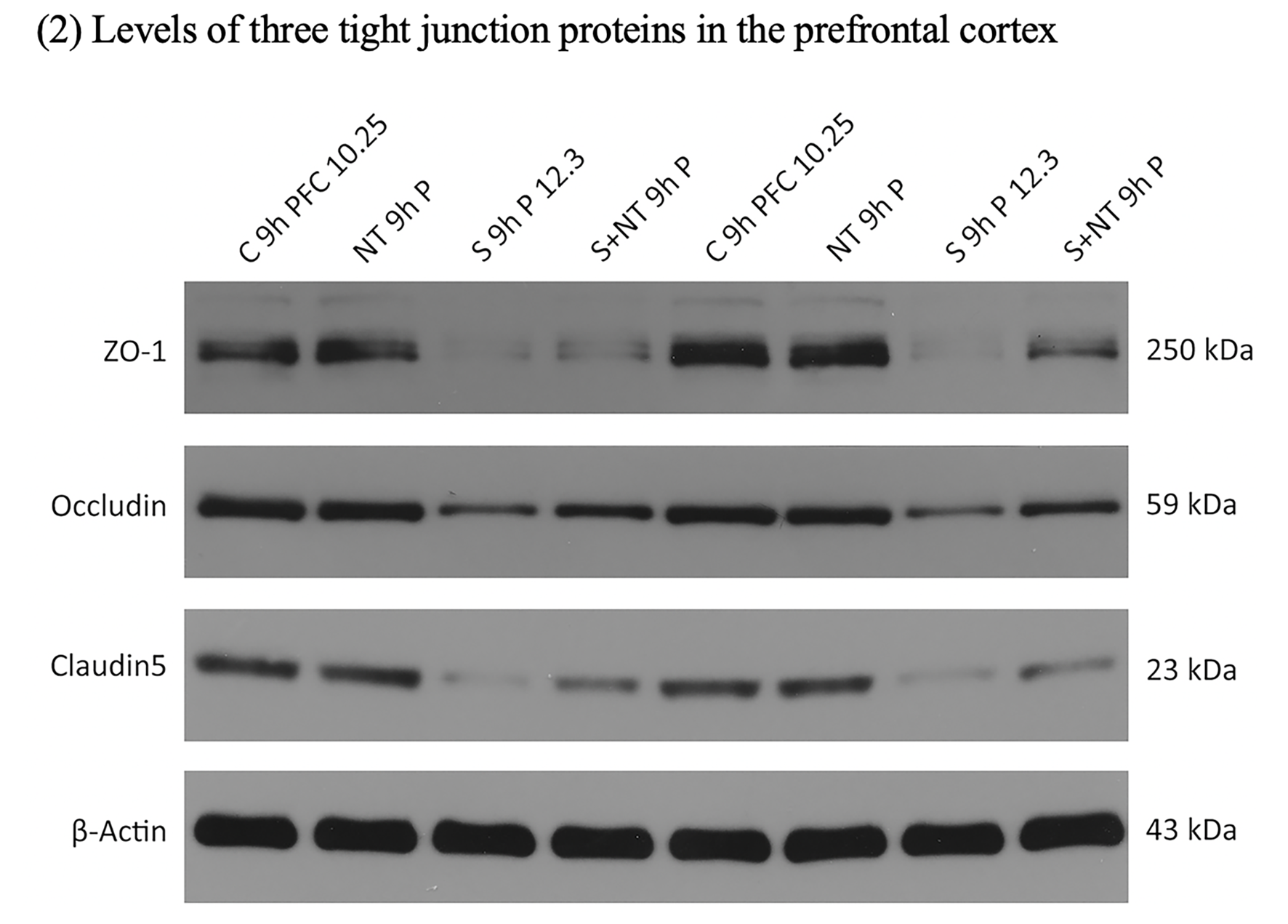

Supplement: Supplementary file 2 [file Image_2.TIF]

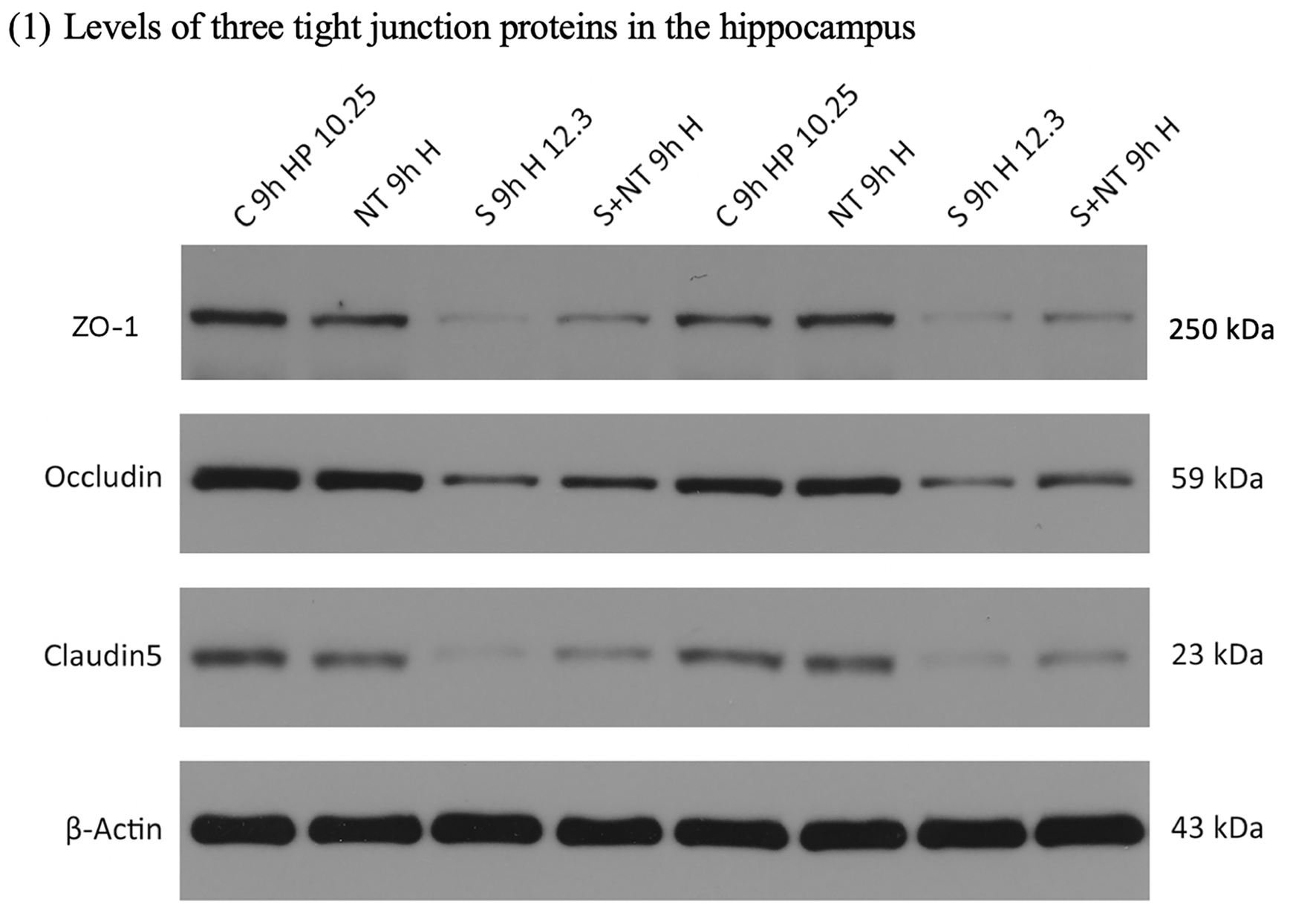

Supplement: Supplementary file 3 [file Image_3.TIF]
